# Misdiagnosed gastrinoma: A case report

**DOI:** 10.3892/ol.2014.2019

**Published:** 2014-04-01

**Authors:** QI-KAI SUN, WEI WANG, HANG-CHENG ZHOU, YANG LV, JI-HAI YU, JIN-LIANG MA, WEI-DONG JIA, GE-LIANG XU

**Affiliations:** 1 Department of General Surgery, Anhui Provincial Hospital, Anhui Medical University, Hefei, Anhui 230001, P.R. China; 2 Department of Medical Oncology, Anhui Provincial Hospital, Anhui Medical University, Hefei, Anhui 230001, P.R. China; 3 Department of Pathology, Anhui Provincial Hospital, Anhui Medical University, Hefei, Anhui 230001, P.R. China

**Keywords:** gastrinoma, neuroendocrine tumor, solid pseudopapillary tumor of pancreas, misdiagnosis

## Abstract

Gastrinoma is most commonly located in the gastrinoma triangle (comprising of the duodenum, pancreas and bile ducts) or in the adjacent lymph nodes. Due to the low mortality rate, it is often misdiagnosed as other diseases with similar clinical characteristics, such as a solid pseudopapillary tumor of the pancreas (SPTP). Therefore, the current study reports a rare case of gastrinoma located in the tail of the pancreas of a female patient under medical examination, who exhibited no clinical symptoms. The tumor, which was located in the body and tail of the pancreas, was successfully resected and the spleen was preserved. The outcome of surgery combined with the postoperative pathological examination resulted in the patient being misdiagnosed with a SPTP. During the consequent six-year follow-up period, low-density liver lesions and an intractable peptic ulcer gradually appeared. Finally, the patient diagnosis was confirmed as a malignant pancreatic neuroendocrine carcinoma with liver metastases. On June 1, 2011, a liver transplant was successfully performed and the patient has maintained a good overall condition. The underlying clinical and pathological factors that may have resulted in misdiagnosis are investigated in the present study. Through providing our preliminary clinical experiences and lessons, the aim of the present study was to focus the attention of clinicians on this type of cancer in order to improve its diagnosis and treatment.

## Introduction

Gastrinoma is a type of neuroendocrine tumor with an incidence of approximately one to three cases per year for every 1,000,000 individuals (1). At present, ~50–60% of gastrinoma cases are malignant (2) and a five-year survival rate of patients with metastatic gastrinoma is possible for 20–40% of these cases (3). To the best of our knowledge, 90% of gastrinomas are located in the gastrinoma triangle comprising the duodenum (60–80%) and the pancreas (10–40%), or in the adjacent lymph nodes (1). The first common signs of gastrinoma include secretory diarrhea, peptic telephium, severe esophagitis and hypercalcemia (4). Ectopia and asymptomatic gastrinoma are rarely reported in the literature. In the current study, a rare case report of gastrinoma is presented and the underlying clinical and pathological factors that may have resulted in the misdiagnosis are investigated. The aim of the present study was to focus the attention of clinicians on this type of cancer by providing preliminary clinical experience in order to improve its diagnosis and treatment.

## Case report

**Figure 1:**
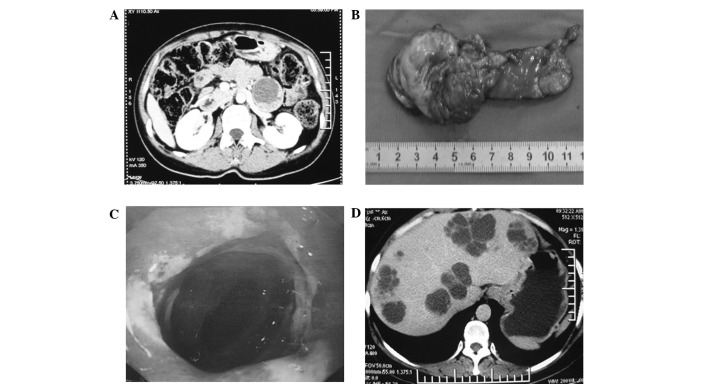
Surgical specimen, and imaging and endoscopic examination results. (A) Enhanced computed tomography (CT) showed a cystic mass in the pancreatic body and tail. (B) Intraoperative resected mass of the pancreatic body and tail. (C) Following 24 months of follow-up, a gastroscopy revealed a duodenal bulbar ulcer and hemorrhagic gastric body inflammation. (D) Enhanced CT scans of the abdomen revealed multiple round lesions located in the left and right lobes of the liver.

**Figure 2:**
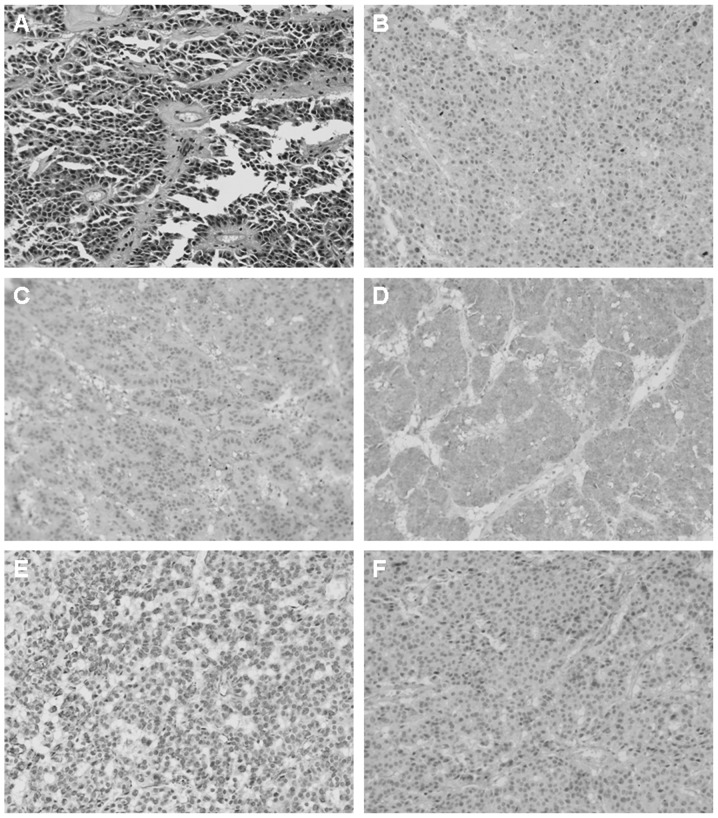
Histopathological and immunohistochemistry results. (A) Surgical removal of mass is shown by the hematoxylin and eosin staining and (B) α1-antichymotrypsin-positive; (C) neuron-specific enolase-positive; (D) synaptophysin-positive; (E) vimentin-positive; and (F) cytokeratin 7-negative staining. Magnification, ×400.

On December 15, 2006, a 39-year-old female was admitted to the Anhui Provincial Hospital (Anhui, China) following the observation of a pancreatic body and a tail lesion in an annual periodic health examination. The patient had not experienced any discomfort and was hemodynamically stable. A benign abdominal examination was conducted and all of the blood and biochemical parameters were within their respective reference ranges. The family and personal medical histories were unremarkable. Written informed consent was obtained from the patient for publication of the present study and any accompanying images. Enhanced computed tomography (CT) revealed a cystic mass in the pancreatic body and tail (Fig. 1A). Considering these observations, the patient was diagnosed with pancreatic body and tail space-occupying lesions. The patient also underwent a distal pancreatectomy (Fig. 1B). A solid pseudopapillary tumor of the pancreas (SPTP) was suspected as a result of postoperative pathology (World Health Organisation, 2010 guideline: Low malignant potential) (5). Immunohistochemistry results indicated that the tumor cells were positive for α1-antichymotrypsin, synaptophysin (Syn), vimentin and neuron-specific enolase (Fig. 2). In the following year, the patient remained well and no apparent symptoms occurred. However, the patient was referred to the Anhui Provincial Hospital after presenting with stomachache in December 2008. After 24 months of follow-up, a gastroscopy revealed a duodenal bulbar ulcer and hemorrhagic gastric body inflammation (Fig. 1C). B-scan ultrasonography of the abdomen also revealed a low-density liver lesion (size, 1 cm). The patient was diagnosed with a peptic ulcer (active stage) and was administered proton pump inhibitor (PPIs; including omeprazole) treatment. Following the second hospitalization, the condition and ulcer symptoms of the patient improved during the follow-up period. Although the ulcer healed, an additional gastroscopy in June 2009 showed gastrointestinal erosion. B-scan ultrasonography revealed that multiple, small low-density liver lesions in the left lobe of the liver. The patient was continuously treated with the PPI (omeprazole). However, the patient rejected further treatment, as the low-density liver lesion was asymptomatic. In October 2010, a gastroscopy demonstrated the presence of an ulcer that was affecting the stomach and duodenum. Although the PPI was continuously administered, the patient exhibited symptoms, including belching and acidic reflux. Enhanced CT scans of the abdomen revealed multiple round lesions located in the left and right lobes of the liver (Fig. 1D). Positron emission tomography-CT showed pancreatic malignant lesions and multiple metastatic neoplasia of the liver. A gastrinoma with liver metastasis was suspected on the basis of the patient’s medical history. The serum gastrin level of the patient (237.97 pg/ml) was abnormal (normal, <100 pg/ml). A new biopsy specimen from the pancreatic body and tail mass (obtained at the Zhongshan Hospital, Fudan University, Shanghai, China) indicated a neuroendocrine carcinoma. Immunohistochemical staining for gastrin, chromogranin (CgA) and Ki-67 was positive. Gastrinoma of the pancreatic body and tail with liver metastases was confirmed. The patient subsequently received treatment with octreotide acetate (20 mg/month) and sunitinib (37.5 mg/day), which had a poor effect. Finally, a liver transplant was successfully performed for the patient on June 1, 2011. The patient currently maintains a good overall condition.

## Discussion

The current study presents a rare case of a female patient with malignant pancreatic neuroendocrine carcinoma with liver metastases, who was initially misdiagnosed with SPTP. The potential underlying clinical and pathological factors that may have led to misdiagnosis are analyzed and listed in the present study.

First, the majority of gastrinomas are located in the gastrinoma triangle (comprising of the duodenum and the pancreas) or in the adjacent lymph nodes (1), whereas the present case of gastrinoma occurred in an unusual location, the pancreatic body and tail, which is outside the typical triangle. Therefore, the location complicated the diagnosis.

Secondly, it is known that gastrohelcosis and diarrhea are common first signs of a gastrinoma, particularly in non-functioning cases, while SPTP are generally characterized in young females with no clinical symptoms prior to the onset of the illness, negative tumor markers and without a history of peptic ulcers (6). Combined with the clinical data of the patient, it is particularly difficult to initially differentiate between gastrinomas and SPTP. This was another significant cause of erroneous diagnosis during the early stage of the disease in the present case.

**Figure 3:**
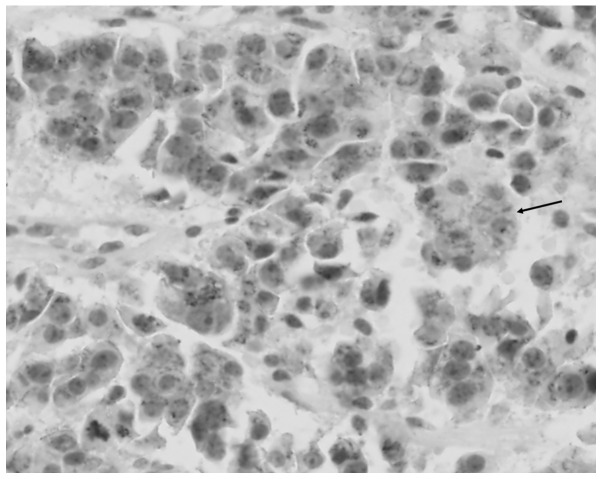
Immunohistochemical result of β-catenin staining. Membrane and cytoplasmic positive expression of the β-catenin protein without nuclear staining (arrow). Magnification, ×400.

Thirdly, pathological assessment is the most reliable diagnostic basis and considered to be the gold standard. However, it is worth noting that the characteristics of hematoxylin and eosin (H&E) staining with gastrinoma and solid pseudopapillary tumors are similar and may be confused, even when specific typical immunohistochemical indicators, such as Syn or CgA (7), are observed. Interstitial hyaline degeneration and false papillary structure are the two microscopically characterized changes of SPTP and corresponding changes are observed in the pathological section when performing H&E staining. Therefore, combined with the clinical data and experiences of the patient, our pathologists were confident in the initial diagnosis of SPTP. However, whether pancreatic neuroendocrine tumors can be diagnosed with any certainty when the abovementioned immunohistochemical indicators are positive remains a controversial issue. The associated literatures on this issue were reviewed and certain studies found that specific SPTP patients were misdiagnosed with pancreatic neuroendocrine tumors even though expression of Syn and CgA was positive (7–11). Thus, the classic index is not completely reliable, however, the studies also found that E-cadherin and β-catenin were potentially effective in the differential diagnosis of the two diseases. Therefore, in the present study the immunostaining of β-catenin was conducted, which supplemented our results and were consistent with previous studies (Fig. 3). However, this conclusion requires support from large-scale clinical trials.

Finally and most significantly, a monism, rather than pluralism, approach is required to diagnose the disease. The patient in the present case experienced intractable recurrent peptic ulcers and multiple low-density liver lesions developed continuously during the postoperative follow-up period. If clinicians are able to connect these novel conditions with previous surgical history, it may allow a correct revised diagnosis to be obtained promptly following surgery.

Therefore, a serious misdiagnosis emphasizes the requirement for a more accurate differential diagnosis of such a rare case of a disease without a typical clinical manifestation. The aim of the present study was to focus the attention of clinicians on this disease in order to improve its diagnosis and treatment to a certain extent.

In conclusion, pancreatic gastrinoma should be carefully differentiated from SPTP during diagnosis. Immunostaining of E-cadherin and β-catenin may be necessary to aid with the differential diagnosis.
